# Prevalence and incidence of autism in children and adolescents in Manitoba, Canada: An updated estimate using population-based administrative health data from 2011 to 2022

**DOI:** 10.17269/s41997-025-01113-6

**Published:** 2025-10-16

**Authors:** Deepa Singal, Jennifer E. Enns, Kevin Friesen, Karen Bopp, Margherita Cameranesi, Ana Hanlon-Dearman, Jonathan Lai, Nathan C. Nickel, Shahin Shooshtari, Lonnie Zwaigenbaum, Marni Brownell

**Affiliations:** 1 Manitoba Centre for Health Policy, Rady Faculty of Health Sciences, University of Manitoba, Winnipeg, MB Canada; 2 Autism Alliance of Canada, North York, ON, Canada; 3 Educational and Counselling Psychology, and Special Education, Faculty of Education, University of British Columbia, Vancouver, BC Canada; 4 Department of Psychology, Saint Mary’s University, Halifax, NS Canada; 5 Department of Pediatrics and Child Health, Rady Faculty of Health Sciences, University of Manitoba, Winnipeg, MB Canada; 6 Institute of Health Policy, Management and Evaluation, Dalla Lana School of Public Health, University of Toronto, Toronto, ON Canada; 7 Department of Community Health Sciences, Rady Faculty of Health Sciences, University of Manitoba, Winnipeg, MB Canada; 8 St. Amant Research Centre, Winnipeg, MB Canada; 9 Department of Pediatrics, University of Alberta, Edmonton, AB Canada

**Keywords:** Adolescent, Child, Autistic disorder, Incidence, Prevalence, Public health, Adolescent, Enfant, Trouble autistique, Incidence, Prévalence, Santé publique

## Abstract

**Objectives:**

Estimates of autism prevalence are critical for informing evidence-based decisions, allocating resources, and developing effective strategies to support autistic individuals and their families. In Canada, such estimates remain limited, with the most recent population-based data on autism prevalence and incidence in Manitoba spanning 2004–2015, underscoring the need for more current data.

**Methods:**

We used linked, whole-population administrative health and clinical data to develop a validated identification algorithm. We determined annual prevalence and incidence rates of autism among Manitoba children and adolescents aged 0–17 from 2011 to 2022, and conducted regression modelling to examine changes over time, adjusting for sex, geography, and socioeconomic variables.

**Results:**

We identified 9396 children and adolescents diagnosed with autism during the study period. The prevalence of autism diagnoses was 0.58% (95% CI 0.55–0.60) in 2011 and 1.67% (95% CI 1.63–1.72) in 2022. The incidence of autism diagnoses was 0.79/1000 (95% CI 0.69–0.90) in 2011 and 3.06/1000 (95% CI 2.87–3.27) in 2022. We found statistically significant year-over-year increases in both prevalence and incidence.

**Conclusions:**

Increasing autism prevalence indicates a pressing public health need for sustained investment in specialized healthcare services and supports that promote the full inclusion of autistic people in society. Strengthening surveillance systems across Canada is essential for generating high-quality population-based data to inform policy development and resource allocation and ensuring the health and social needs of autistic people and their families are met.

**Supplementary Information:**

The online version contains supplementary material available at 10.17269/s41997-025-01113-6.

## RÉSUMÉ

**Objectifs:**

Les estimations de la prévalence de l’autisme sont essentielles pour éclairer les décisions fondées sur les données probantes, l’allocation de ressources et la mise au point de stratégies efficaces pour aider les personnes autistes et leurs familles. Au Canada, de telles estimations sont encore limitées; les données populationnelles les plus récentes sur la prévalence et l’incidence de l’autisme au Manitoba couvrent la période de 2004 à 2015, ce qui souligne le besoin de données plus actuelles.

**Méthodes:**

À l’aide de données administratives cliniques et de santé liées couvrant l’ensemble de la population, nous avons mis au point un algorithme d’identification validé. Nous avons déterminé les taux de prévalence et d’incidence annuels de l’autisme chez les enfants et les adolescents du Manitoba (0 à 17 ans) entre 2011 et 2022 et utilisé des modèles de régression pour étudier leur évolution dans le temps, en apportant des ajustements en fonction du sexe, du lieu géographique et du statut socio-économique.

**Résultats:**

Nous avons identifié 9 396 enfants et adolescents ayant un diagnostic d’autisme au cours de la période de l’étude. La prévalence des diagnostics d’autisme était de 0,58% (IC de 95% 0,55–0,60) en 2011 et de 1,67% (IC de 95% 1,63–1,72) en 2022. L’incidence des diagnostics d’autisme était de 0,79 p. 1000 (IC de 95% 0,69–0,90) en 2011 et de 3,06 p. 1000 (IC de 95% 2,87–3,27) en 2022. Les hausses annuelles étaient significatives tant pour la prévalence que pour l’incidence.

**Conclusions:**

La prévalence croissante de l’autisme est le signe d’un pressant besoin d’investissements de santé publique soutenus dans les services de santé spécialisés et les mesures de soutien qui favorisent l’inclusion complète des personnes autistes dans la société. Il est également essentiel de renforcer les systèmes de surveillance de tout le Canada en vue de produire des données populationnelles de haute qualité pour éclairer l’élaboration de politiques et l’allocation de ressources et nous assurer de répondre aux besoins sociaux et de santé des personnes autistes et de leurs familles.

## Introduction

Autism (also known as autism spectrum disorder or ASD) is a neurodevelopmental condition characterized by differences in social interaction, communication, sensory processing, and behaviour, with considerable variation among individuals (American Psychiatric Association (APA) DSM-5 Task Force, 2022). Co-occurring conditions, including intellectual/learning disabilities, attention-deficit hyperactivity disorder, psychiatric disorders, and/or motor developmental disorders, are also often present in autistic people (Lord et al., 2018; Simonoff et al., 2008) (Box 1). Autism is among the most common and fastest growing neurodevelopmental conditions in Canada. In 2018, the Public Health Agency of Canada (PHAC) estimated that 1 in 66 (1.5%) children and youth aged 5–17 had been diagnosed with autism (Public Health Agency of Canada, 2018). In a subsequent report using data from the Canadian Health Survey on Children and Youth (2022), PHAC estimated that 1 in 50 (2.0%) children and youth aged 1–17 years were autistic (Public Health Agency of Canada, 2022). By comparison, in the United States, the Centers for Disease Control and Prevention reported a prevalence of 1 in 36 (2.8%) among 8-year-old children in 2020 (Maenner et al., 2023), and recent US studies suggest diagnostic rates continue to rise. Increased awareness, improved diagnostic practices, and greater access to developmental assessment are often cited as contributing factors (Mikami et al., 2019).

Box 1. Language Statement

**Table:** 

Language used to describe autism continues to evolve. While some individuals prefer person-first language (e.g., “person with autism”) to emphasize their personhood, many autistic self-advocates use identity-first language (e.g., “autistic person”) to affirm autism as an integral part of their identity. In this paper, we use identity-first language in alignment with the preferences of many in the autistic community, while recognizing that individuals identify in diverse ways.
See the Language Guide from the Autism Alliance of Canada for more information (Autism Alliance of Canada, 2024).

In Canada, access to essential support services, including timely diagnosis, early interventions, educational supports, and specialized healthcare, has not kept pace with the increase in autism prevalence (Malik-Soni et al., 2022). The healthcare system continues to face capacity constraints, creating significant barriers for autistic people and their families. These service gaps are compounded by persistent inequities in access based on geography, socioeconomic status, and availability of care providers with autism expertise (Canadian Academy of Health Sciences, 2022). Public funding remains limited for many services that are vital to health and developmental outcomes, including occupational therapy, speech-language pathology, behavioural therapy, and mental health supports. As a result, families are often forced to pay out of pocket for private services, which can be prohibitively expensive. There are also considerable out-of-pocket costs for family members seeking respite care and costs of productivity loss for autistic people and their families (Rogge & Janssen, 2019). Public investments in supports that promote the health and wellbeing of autistic people and their caregivers remain inadequate (Canadian Academy of Health Sciences, 2022).

Prevalence and incidence estimates for autism are critical to inform the planning and funding of services and supports across the lifespan (Canadian Autism Spectrum Disorders Alliance, 2020). These data help identify areas of greatest need, guide equitable resource allocation, and support the development of policies that promote inclusion and health equity. The growing number of autistic individuals in Canada signals an urgent public health need for expanded and coordinated support systems. However, Canada lacks current, high-quality, population-based data on autism prevalence and incidence at the provincial and territorial levels, an essential foundation for evidence-informed decision making. Although the 2018 PHAC report was an important first step, it excluded several provinces and territories, including Manitoba, Alberta, and Saskatchewan (Public Health Agency of Canada, 2018). The 2019 Canadian Health Survey on Children and Youth included all ten provinces and three territories, but the data are cross-sectional and limited by a 52.1% response rate; they also do not include information about children in care of Child Protection Services or Indigenous children living on reserves (Public Health Agency of Canada, 2022).

Given that health and education systems in Canada are governed provincially, timely and robust data at the provincial level are crucial to ensuring that services meet the needs of autistic individuals and their families. In Manitoba, the most recent population-based longitudinal data are from 2004 to 2015, during which time autism rates increased substantially in early childhood (Hamad et al., 2019). The current study expands on this work with data through to 2022 and ascertains the prevalence and incidence of autism in early childhood, school-aged children, and adolescents up to age 17 years. Our study is particularly timely given the release of Canada’s first National Autism Strategy in 2024 (Public Health Agency of Canada, 2024), which identifies research, data, and surveillance as key priorities. Up-to-date, high-quality data are foundational for evidence-informed policy and planning to improve health equity and ensure autistic individuals across Canada have access to the supports and services they need to thrive.

## Methods

### Study setting

Manitoba is a central Canadian province with a population of 1.4 M. Approximately 72% of the population lives in urban settings (mainly in Winnipeg and Brandon), while the remainder live in smaller rural or remote communities (Statistics Canada, 2025). Like all Canadian provinces and territories, Manitoba has a universal public health insurance plan; medically necessary primary care and hospital care is offered to residents without requiring out-of-pocket payment. Autism services and supports available to individuals and families vary by region. However, unique to Manitoba is their comprehensive data repository comprising linkable administrative, clinical, and registry data, which makes it possible to conduct whole-population studies of residents’ health and health services use (Katz et al., 2020). The repository also includes data on most children in care and Indigenous families living on reserves. In this study, we draw on the strengths of these data to determine the prevalence and incidence of autism in a population-based cohort of Manitobans and examine time trends by age, sex, income, and urban/rural geography.

### Data sources

The study was conducted using the Population Research Data Repository housed at the Manitoba Centre for Health Policy (MCHP), University of Manitoba (Katz et al., 2020; Smith et al., 2015). The repository contains whole-population administrative, registry, survey, and other types of data on virtually all (> 99.9%) residents of Manitoba through contacts with the universal health system, social services, public education system, and/or justice system. All of the repository records are de-identified (names and addresses removed), but individual-level linkage across databases is achieved through scrambled Personal Health Identification Numbers (PHINs) attached to each record. The specific databases used in this study include the Manitoba Health Insurance Registry, which captures sociodemographic information for all Manitobans eligible to receive health services and is the central registry used for linkage via the scrambled PHINs; hospital discharge abstracts, which contain data on in-patient hospitalizations, diagnoses, and procedures; physician billing claims, which capture ambulatory visits to primary care providers and specialist physicians; and data on clinical diagnoses of autism from 2011 to 2022 from the Rehabilitation Centre for Children, a community-based health centre in Winnipeg, Manitoba, providing specialized rehabilitation, developmental and diagnostic programs and services to children and youth with a variety of health diagnoses and conditions, including autism. We also accessed the public use Canada Census files through Statistics Canada to examine small area-level income data.

### Administrative data definition of autism

The use of administrative data to identify autism has gained traction in recent years due to cost-effectiveness and the ability to capture large, diverse populations (Dodds et al., 2009). Several different algorithms using a combination of health service use, educational support, and social services data have been applied in Canadian studies (O’Donnell et al., 2022). Our case definition is based on the International Classification of Disease (ICD) coding system (9th and 10th revisions) and has been tested and validated against clinical diagnostic data from the Rehabilitation Centre for Children with a sensitivity of 77.4%. We defined autism as having one or more hospitalizations with an ICD-10-CA code of ICD-F84.0 (childhood autism), F84.1 (atypical autism), F84.2 (Rett syndrome), F84.3 (other childhood disintegrative disorder), F84.4 (overactive disorder associated with mental retardation and stereotyped movements), F84.5 (Asperger’s syndrome), F84.8 (other pervasive developmental disorders), or F84.9 (pervasive developmental disorder, unspecified), or two or more physician visits within 2 years with an ICD-9 code of 299 (pervasive developmental disorder).

### Cohort development

**Fig. 1:**
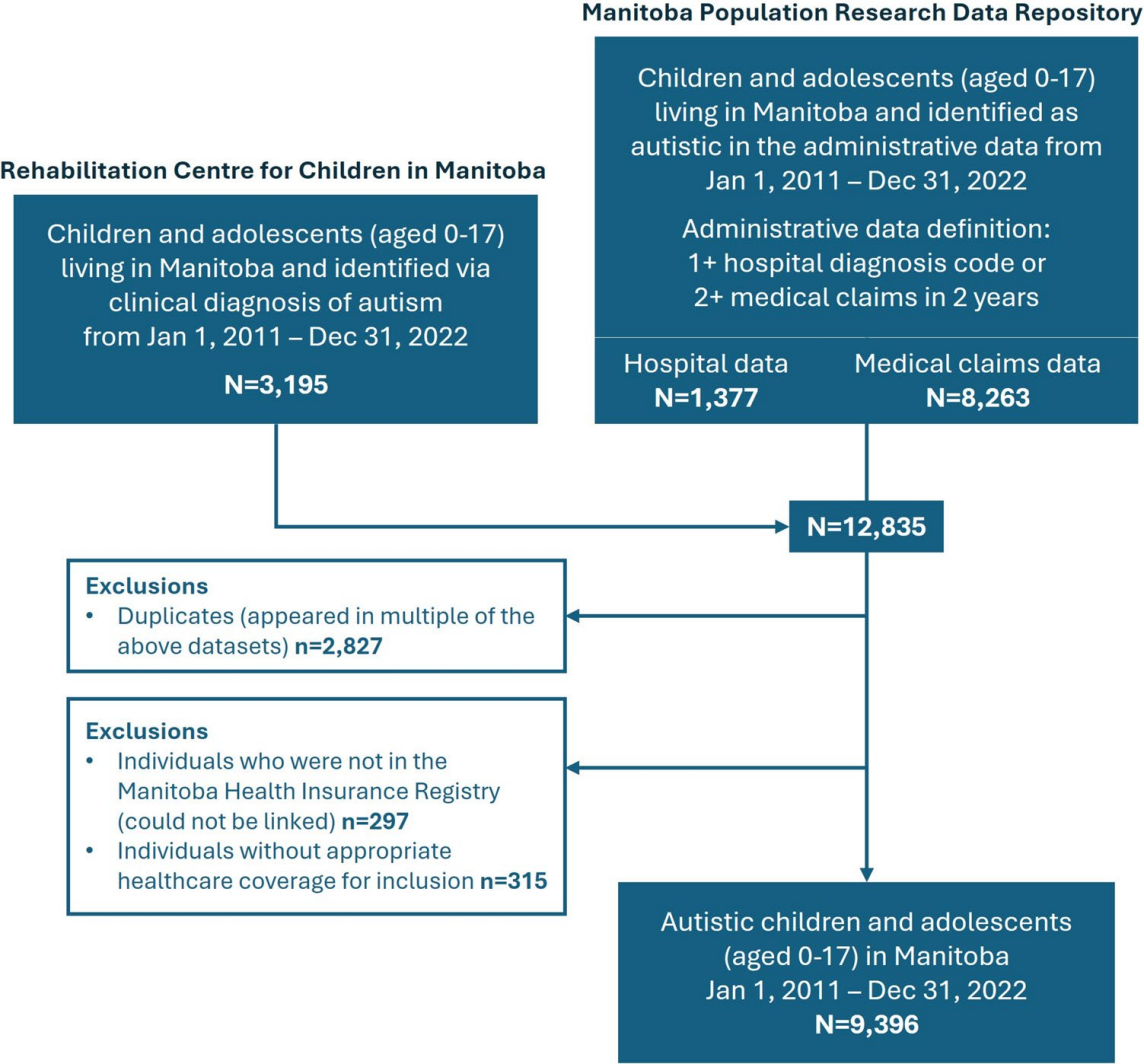
Cohort creation flow diagram

The development of the study cohort is depicted in Fig. 1. Autism cases among children and adolescents aged 0–17 years from 2011 to 2022 were identified in clinical data from the Rehabilitation Centre for Children and administrative data in the repository (hospital and medical claims data). We removed duplicates (individuals that appeared in more than one of these databases), individuals with a clinical diagnosis whose data could not be linked to the repository (e.g., because of missing information), and individuals who did not have healthcare coverage during the study period.

### Analysis

To create the income quintiles, six-digit postal codes from the Manitoba Health Insurance Registry were linked to dissemination areas (population of 400–700 persons) in the Census file; each person living in the dissemination area was attributed the average household income value from the Census. This yielded neighbourhood or small area-level incomes, which were then ordered from lowest to highest and divided into five groups with approximately 20% of the population in each group (i.e. in each quintile) (Manitoba Centre for Health Policy, 2008).

Crude annual autism prevalence and incidence rates were calculated for the overall cohort and by age group (0–5, 6–12, and 13–17 years), sex (male/female sex assigned at birth), and urban and rural income quintiles (Q1–Q5). For prevalence, the numerator was the number of individuals aged 0–17 years with an autism diagnosis during the calendar year; the denominator was the average number of individuals aged 0–17 years living in Manitoba in each year. For incidence, the numerator was the number of new autism diagnoses in each calendar year among individuals aged 0–17 years; the denominator was the total person-years contributed by individuals aged 0–17 years who were not previously diagnosed with autism. To determine whether these rates changed over time, we used Poisson regression (prevalence) and negative binomial regression (incidence per 1000 people), adjusting for sex, geography, and income. The models generated relative rates to estimate year-over-year changes in autism prevalence and incidence. All analyses were conducted using SAS statistical analysis software Version 9.4.

### Ethics

The study received approvals from the University of Manitoba’s Health Research Ethics Board (HS25757–H2022:364), the Provincial Health Research Privacy Committee (No. P2022-126), and the data providers, including the Manitoba Ministry of Health and Long-Term Care and the Rehabilitation Centre for Children in Winnipeg, Manitoba.

## Results

### Annual prevalence and incidence of autism

**Fig. 2:**
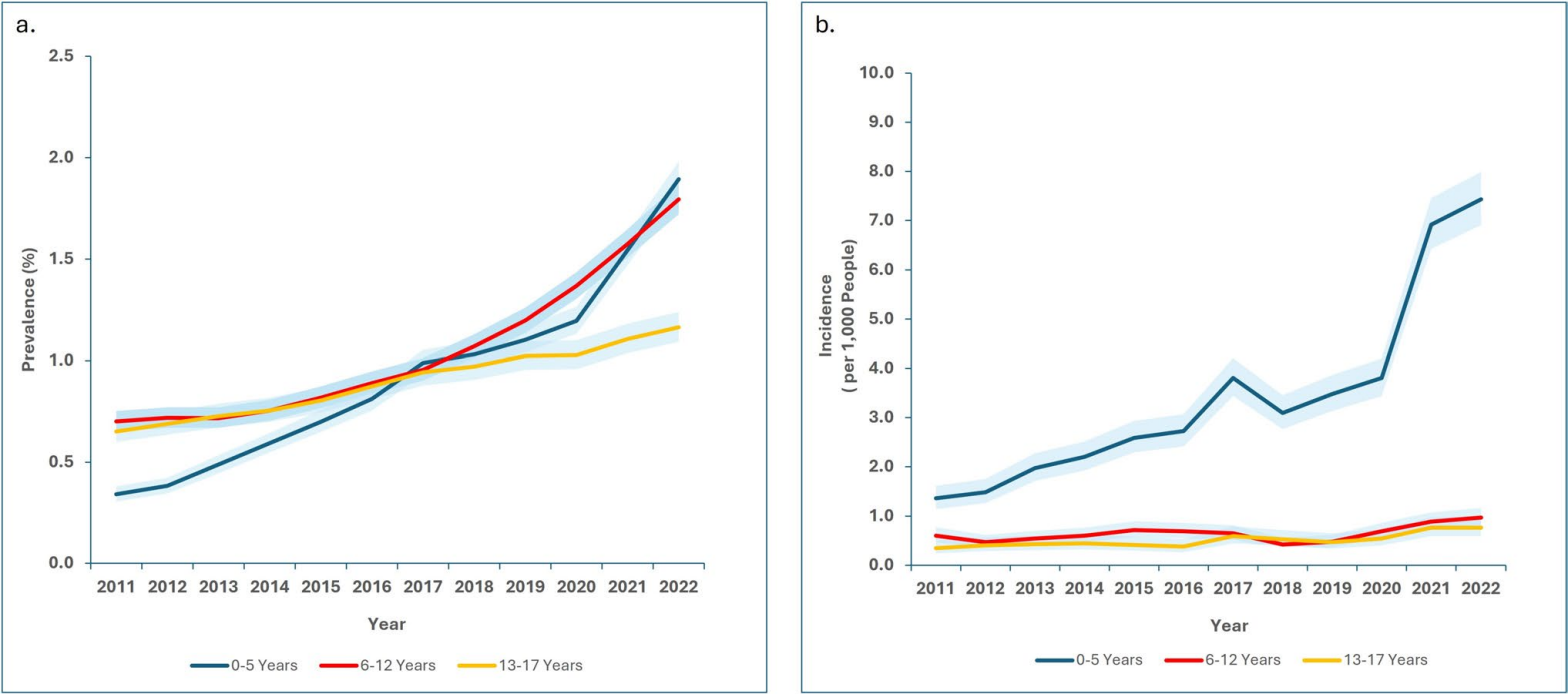
Crude annual prevalence (**a**) and incidence (**b**) of autism in Manitoba children and adolescents aged 0–17 years, by age group (2011–2022)

**Fig. 3:**
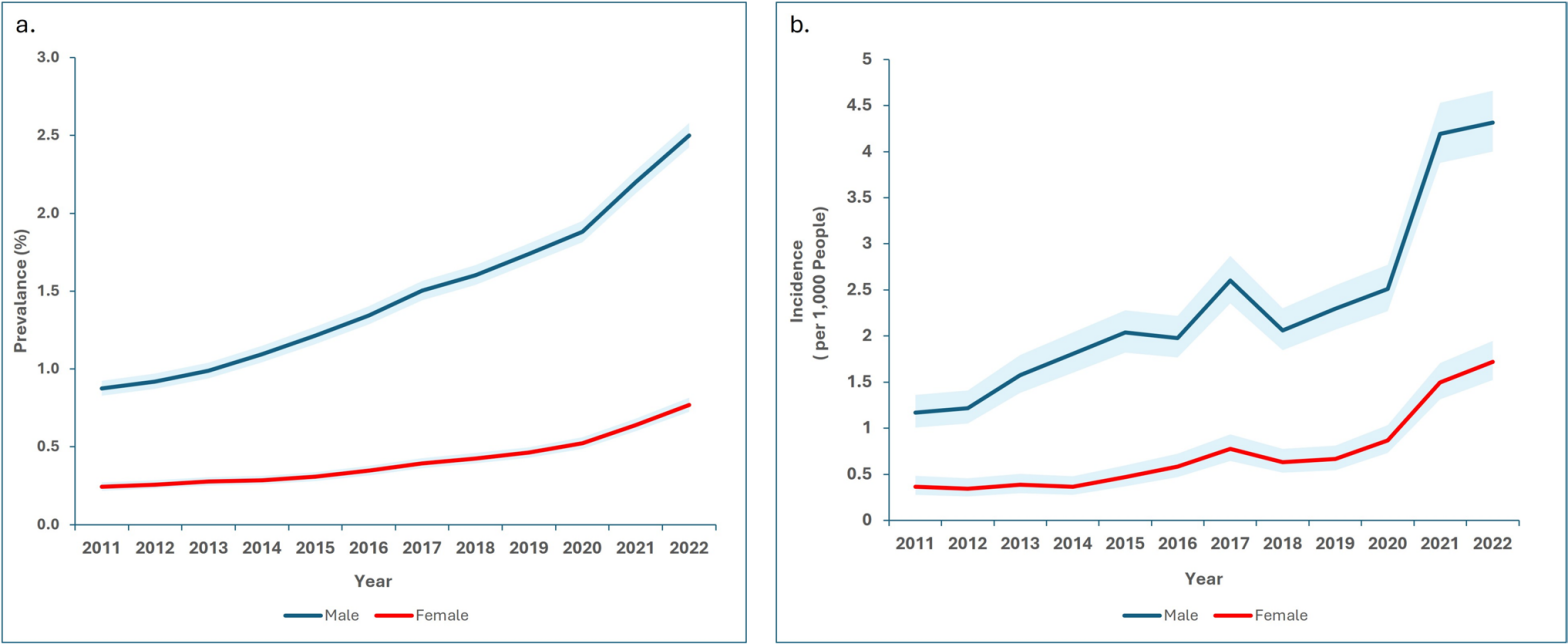
Crude annual prevalence (**a**) and incidence (**b**) of autism in Manitoba children and adolescents aged 0–17 years, by sex (2011–2022)

The final study cohort comprised 9396 individuals aged 0–17 years. Annual prevalence and incidence rates from 2011 to 2022 are shown in Table 1. The crude prevalence of autism increased by a factor of 2.9 from 2011 (0.58%, 95% CI 0.55–0.60) to 2022 (1.67%, 95% CI 1.63–1.72). The crude incidence increased by a factor of 3.9 from 2011 (0.79 new diagnoses/1000 people, 95% CI 0.69–0.90) to 2022 (3.06 new diagnoses/1000 people, 95% CI 2.87–3.27). Stratifying the cohort by age group (Fig. 2) revealed that the increase in prevalence was mainly driven by the younger age groups (0–5 years and 6–12 years), and the increase in incidence was mainly due to the youngest age group (0–5 years). Stratifying by sex (Fig. 3) demonstrated that in 2022, autism was 3.3 times more prevalent in males than females, and 2.5 times more males than females received a new diagnosis that year. The annual numeric estimates and confidence intervals for these two figures can be found in Appendix 1 and 2. Additional strata showing crude prevalence and incidence rates by urban and rural income quintiles are presented in Appendix 3. In both urban and rural areas, the lowest income quintiles generally had a higher prevalence and incidence of autism than the higher income quintiles, although rates in all income groups, regardless of geography, rose over time.

**Table 1:** Annual prevalence and incidence of autism in Manitoba from 2011 to 2022. Children and adolescents aged 0–17 years

Prevalence				
Year	Total population	Autism cases	Autism prevalence (%)	95% CI
2011	286,849	1653	0.58	0.55, 0.60
2012	291,476	1770	0.61	0.58, 0.64
2013	293,138	1918	0.65	0.63, 0.68
2014	294,586	2097	0.71	0.68, 0.74
2015	296,612	2331	0.79	0.75, 0.82
2016	299,218	2598	0.87	0.84, 0.90
2017	303,210	2956	0.97	0.94, 1.01
2018	306,057	3191	1.04	1.01, 1.08
2019	306,843	3473	1.13	1.09, 1.17
2020	308,664	3807	1.23	1.19, 1.27
2021	308,335	4479	1.45	1.41, 1.50
2022	309,448	5174	1.67	1.63, 1.72
Incidence				
Year	Total person-years	Newly diagnosed autism cases	Autism diagnosis rate/1000 people	95% CI
2011	284,087	223	0.79	0.69, 0.90
2012	288,139	229	0.79	0.70, 0.90
2013	288,895	296	1.02	0.91, 1.15
2014	290,120	319	1.10	0.99, 1.23
2015	291,950	372	1.27	1.15, 1.41
2016	295,293	386	1.31	1.18, 1.44
2017	297,957	511	1.72	1.57, 1.87
2018	300,006	410	1.37	1.24,1.24
2019	301,042	455	1.51	1.38, 1.51
2020	303,392	521	1.72	1.58, 1.87
2021	301,833	868	2.88	2.69, 3.07
2022	302,409	926	3.06	2.87, 3.27

*CI*, confidence interval

### Changes over time

The results of the regression models (time trends) for prevalence and incidence are shown in Table 2. For the overall cohort, the relative prevalence rate was 1.103, indicating a 10.3% year-over-year increase in autism prevalence over the study period. The relative rate for the younger age groups was not different from the 13–17-year age group. For female children, the relative prevalence rate was lower than for male children (relative rate 0.276). The relative incidence rate for the overall cohort was 1.122, indicating a 12.2% year-over-year increase in autism incidence over the study period. The relative incidence rates for both the younger age groups (and especially the 0–5-year age group) were higher than for the oldest age group, and for female children, the relative incidence rate was lower than for male children (relative rate 0.313). The results of the regression models for the urban/rural income strata are shown in Appendix 4. As with the crude rates, the relative increases in prevalence and incidence of autism in urban areas were driven mainly by the lower-middle income quintiles (Q1–Q3); in rural areas, relative rates increased a similar amount in all quintiles.

**Table 2:** Annual trends in autism prevalence and incidence in Manitoba from 2011 to 2022. Children and adolescents aged 0–17 years, by age group and sex

Prevalence			
		Estimate	95% CI
Overall	Relative rate (difference each year)	1.103	1.100, 1.106
By age group	Relative rate (difference each year)	1.100	1.085, 1.115
	Age 0–5	0.917	0.815, 1.030
	Age 6–12	1.106	0.985, 1.242
	Age 13–17 (ref group)	1.000	1.000, 1.000
By sex	Relative rate (difference each year)	1.104	1.100, 1.107
	Female	0.276	0.269, 0.283
	Male (ref group)	1.000	1.000, 1.000
Incidence			
		Estimate	95% CI
Overall	Relative rate (difference each year)	1.122	1.097, 1.148
By age group	Relative rate (difference each year)	1.090	1.066, 1.114
	Age 0–5	6.048	5.001, 7.313
	Age 6–12	1.278	1.048, 1.560
	Age 13–17 (ref group)	1.000	1.000, 1.000
By sex	Relative rate (difference each year)	1.129	1.120, 1.138
	Female	0.313	0.294, 0.334
	Male (ref group)	1.000	1.000, 1.000

*CI*, confidence interval

## Discussion

This study provides updated population-based estimates of autism prevalence and incidence in Manitoba children and adolescents. Using administrative health data from 2011 to 2022, we found that both incidence and prevalence of diagnosed autism have continued to rise steadily over the past decade. In 2022, the prevalence rate of autism among individuals aged 0–17 years in Manitoba was 1.67%, with a relative increase of 10.3% each year since 2011. The incidence rate in 2022 was 3.06 per 1000 people, with a relative annual increase of 12.2% since 2011. Most new diagnoses were among 0–5-year-olds, and consistent with the literature, autism was diagnosed more frequently among male children than female children—specifically, in our most recent year of data (2022), there were 2.5 times more new diagnoses in male than female children and adolescents.

Compared to previously published Manitoba-specific estimates from 2004 to 2015 (Hamad et al., 2019), which demonstrated that rates of autism diagnosis in early childhood were increasing, our updated analysis confirms that this trend has continued and has even accelerated over the past decade. The earlier Manitoba study found relative annual increases of 1.69 (95% CI 1.56–1.83) in prevalence and 1.84 (95% CI 1.62–2.09) in incidence among children aged 1–5 years (Hamad et al., 2019). Our study extends this work through 2022 and across the full 0–17 year age range. Our findings also reflect increasing diagnostic activity in younger children, particularly among those aged 0–5 years, and persistent sex-based disparities in diagnosis, with males more likely to be identified. Together, these data highlight a sustained and growing public health demand that will require coordinated provincial action to ensure early identification, timely access to services, and appropriate support throughout the lifespan. Our finding that autism prevalence is on the rise also aligns with previously published national and international prevalence data. The 2018 PHAC report, which used data from 2015, presented provincial prevalence rates ranging from 0.8% (Yukon) to 1.9% (New Brunswick) with a national average estimate of 1.5% (Public Health Agency of Canada, 2018). In the 2022 PHAC report, which was based on data from the 2019 Canadian Health Survey of Children and Youth, prevalence ranged from 0.8% (Saskatchewan) to 4.1% (New Brunswick) with a national average of 2.0% (Public Health Agency of Canada, 2022). Although these rates are not directly comparable to our current population-based estimates, they confirm the continued upward trend in autism diagnoses nationally. The rates we calculated are consistent with, or slightly below, national average levels—but are increasing steadily over time. Recent studies from the USA provide further support for our findings, particularly with respect to the increasing rate of diagnosis in older age groups. A large cross-sectional analysis of US data from 2011 to 2022 found that the relative increases in diagnosis rates were highest among adolescents and young adults (Grosvenor et al., 2024). This trend has critical implications for health system planning, as it suggests that many autistic people are not being identified until later in childhood or adolescence. In Manitoba and Canada, where comprehensive and integrated services for youth transitioning to adulthood are limited, this shift should be a flag for health, social, and education system planners. Ensuring that services are flexible and responsive across the lifespan is essential to mitigating the risks of poor outcomes in education, employment, and mental health for late-diagnosed individuals (Canadian Autism Spectrum Disorders Alliance, 2020; Public Health Agency of Canada, 2024).

Several factors likely contribute to the rising rates of autism diagnosis observed in these populations. Increased public awareness, enhanced training of clinicians and educators, and improvements in developmental screening and diagnostic practices have likely led to earlier and more frequent identification (Zwaigenbaum et al., 2019). The expansion of diagnostic criteria over time, as well as greater recognition of the autism spectrum in both clinical and community settings, may also explain part of the observed increase (Rosen et al., 2021). Additionally, there is growing understanding of autism presentation across genders and among individuals with co-occurring conditions, which may be contributing to broader identification in historically underdiagnosed groups (Sauer et al., 2021; Simcoe et al., 2023).

However, it must be clearly stated that each of the estimates presented here is very likely under-representative of the true prevalence and incidence of autism for several reasons. First, our findings are based on administrative health data, which identify individuals who have accessed healthcare services and received a formal autism diagnosis. Undiagnosed individuals are not recorded, and this likely results in underestimation of true prevalence and incidence. People diagnosed through private psychologists may also be missed due to this data not being captured in the repository. Autism also remains underdiagnosed in certain populations, particularly among females, individuals living in rural or remote regions with limited healthcare access, and those from marginalized communities. In Manitoba, approximately 28% of the population resides in rural or fly-in communities (Statistics Canada, 2025), where specialized diagnostic and therapeutic services are often limited; thus, these individuals may be missing from the administrative data. In our study, we observed lower prevalence and incidence rates in rural and/or remote regions, but this likely reflects service access limitations rather than true differences in population prevalence.

Second, administrative health data rely on physician diagnostic coding practices, which are influenced by evolving clinical guidelines. There have been substantial revisions to the American Psychological Association diagnostic criteria for autism over the past few decades. The latest versions of the Diagnostic and Statistical Manual of Mental Disorders (DSM-5 and DSM-5-TR) consolidate previously separate categories (e.g., autistic disorder, Asperger’s syndrome) into a single spectrum disorder (autism spectrum disorder) (Rosen et al., 2021). While this reflects the growing recognition that autism presents along a continuum (American Psychiatric Association & DSM-5 Task Force, 2022), it may lead to inconsistencies in diagnostic recording within administrative databases. When diagnoses are made in preschool children, clinicians may use generalized ASD codes without specifying the severity or subtype, which can contribute to cross-disorder diagnostic overlap, particularly with other neurodevelopmental conditions such as ADHD (O’Donnell et al., 2022). These challenges may reduce the specificity of autism coding in health databases. To address these limitations, we used a validated algorithm that draws on multiple sources of administrative data, as recommended by O’Donnell et al. (2022), to increase sensitivity with only a slight cost to the specificity and positive predictive value. However, the accuracy of administrative data identification algorithms can still vary regionally, based on the availability of diagnostic services and specialized care (Brooks et al., 2021).

Finally, although administrative data from the education system can provide additional insight into autism identification, we excluded it from our case algorithm due to recent changes in Manitoba’s school funding model. Prior to 2017/2018, funding for students with additional needs was based on individual applications; however, since then it has been allocated at the school division level through a formula-based model. This change precludes consistent identification of students receiving autism-related support across the full study period and would reduce the comparability and future utility of our algorithm if we included it.

Despite these limitations, our study provides a valuable and policy-relevant update to autism prevalence and incidence in Manitoba. By drawing on a decade of longitudinal, population-based data, our findings contribute to a stronger public health evidence base to inform planning, funding, and service delivery. Several important areas of research remain understudied, including, for example, autism prevalence rates among children in care of Child Protection Services and among Indigenous children. Manitoba has very high rates of children in care compared to other provinces, many of whom are First Nations or Red River Métis (Milne et al., 2023). Accessing these data requires meaningful partnerships with Indigenous researchers and data governance bodies to ensure respect for data sovereignty and Nations-based (Love et al., 2022). Future research led by Indigenous scholars and organizations is essential to better understand autism in these populations.

## Conclusion

This study provides updated population-based estimates of autism prevalence and incidence in Manitoba, revealing a consistent rise from 2011 to 2022. Our reported rates are likely still an underestimation of autism in the population as they only reflect individuals diagnosed through the public healthcare system and exclude those who remain undiagnosed, are diagnosed through private clinicians, or face barriers to accessing care. From a public health perspective, our findings demonstrate that autistic individuals represent a substantial and growing segment of the population whose needs must be addressed through coordinated, accessible, and inclusive health and social systems. Accurate region-specific data are essential to inform evidence-based care planning, service delivery, and equitable resource allocation, both provincially and nationally. As the Federal Government implements its National Autism Strategy, strengthening autism surveillance and addressing service gaps must remain key priorities. Future work should focus on improving data integration across sectors, enhancing representation of underserved populations, and supporting community- and Nations-led research to promote health equity, ensuring that all autistic individuals have the opportunity to thrive.

## Contributions to knowledge

What does this study add to existing knowledge?

- Our study provides updated population-based estimates of autism prevalence and incidence in Manitoba children and adolescents, revealing a consistent rise from 2011 to 2022. In the most recent year available (2022), the prevalence of autism among 0–17-year-olds was 1.67%, with a relative annual increase of 10.3% since 2011, and the incidence was 3.06 per 1000 people, with a relative annual increase of 12.2% since 2011. Most new diagnoses were among 0–5-year-olds, and in 2022, there were 2.5 times more new diagnoses in male than female children and adolescents.

What are the key implications for public health interventions, practice, or policy?

- From a public health perspective, our findings demonstrate that autistic individuals represent a substantial and growing segment of the population whose needs must be addressed through coordinated, accessible, and inclusive health and social systems. Accurate region-specific data are essential to inform evidence-based care planning, service delivery, and equitable resource allocation, both provincially and nationally. As the Federal Government implements its National Autism Strategy, strengthening autism surveillance and addressing service gaps must remain key priorities.

## Electronic supplementary material

Below is the link to the electronic supplementary material.

Supplementary file1 (DOCX 378 KB)

## Data Availability

The source data used in this study were originally collected during the routine administration of health services in Manitoba. They were provided to the Manitoba Centre for Health Policy (MCHP) for secondary use in research under specific data sharing agreements between the data trustees and MCHP. The data are approved for use at MCHP only. They are not owned by the researchers or by MCHP and cannot be deposited in a public repository. However, they are available upon reasonable request. To review source data specific to this article or project, interested parties should contact the MCHP Repository Access & Use team at MCHP.Access@umanitoba.ca. The team will then facilitate data access by seeking the consent of the original data holders and the required privacy and ethics review bodies.

## Appendices

1. Crude Annual Prevalence and Incidence Rates of Autism in Manitoba Children and Adolescents, by Age Group (2011-2022): Numeric estimates corresponding to Fig. 2
2. Crude Annual Prevalence and Incidence Rates of Autism in Manitoba Children and Adolescents, by Age Group (2011-2022): Numeric estimates corresponding to Fig. 3
3. Annual Trends in Autism Prevalence and Incidence in Manitoba from 2011-2022: Children and adolescents aged 0-17 years, by Urban and Rural Income Quintiles

## Code availability

N/A.
