# Supplementary material for: Prevalence and incidence of autism in children and adolescents in Manitoba, Canada: An updated estimate using population-based administrative health data from 2011 to 2022
Source: Can J Public Health. 2025 Oct 16;117(3):554–63. doi: 10.17269/s41997-025-01113-6 (PMC13337990; doi:10.17269/s41997-025-01113-6)
Supplement: Supplementary file 1 — Supplementary file1 (DOCX 378 KB) [file 41997_2025_1113_MOESM1_ESM.docx]

| **Appendix 1. Crude Annual Prevalence and Incidence Rates of Autism in Manitoba Children and Adolescents, by Age Group (2011-2022)**  Numeric estimates corresponding to Figure 2 | | | | | |
| --- | --- | --- | --- | --- | --- |
| **Prevalence** | | | | | |
| **Age group** | **Year** | **Total**  **population** | **Autism cases** | **Autism prevalence (%)** | **95% CI** |
| **0-5 years** | 2011 | 57,917 | 283 | 0.49 | 0.43, 0.55 |
|  | 2012 | 60,463 | 331 | 0.55 | 0.49, 0.61 |
|  | 2013 | 61,895 | 412 | 0.67 | 0.60, 0.73 |
|  | 2014 | 62,399 | 511 | 0.82 | 0.75, 0.89 |
|  | 2015 | 62,972 | 603 | 0.96 | 0.88, 1.04 |
|  | 2016 | 63,547 | 714 | 1.12 | 1.04, 1.21 |
|  | 2017 | 64,483 | 875 | 1.36 | 1.27, 1.45 |
|  | 2018 | 65,551 | 942 | 1.44 | 1.35, 1.53 |
|  | 2019 | 66,108 | 1,031 | 1.56 | 1.47, 1.66 |
|  | 2020 | 66,298 | 1,109 | 1.67 | 1.57, 1.77 |
|  | 2021 | 66,591 | 1,382 | 2.08 | 1.97, 2.19 |
|  | 2022 | 66,404 | 1,733 | 2.61 | 2.49, 2.74 |
| **6-12 years** | 2011 | 99,993 | 731 | 0.73 | 0.68, 0.79 |
|  | 2012 | 101,434 | 761 | 0.75 | 0.70, 0.81 |
|  | 2013 | 103,039 | 771 | 0.75 | 0.70, 0.80 |
|  | 2014 | 105,471 | 824 | 0.78 | 0.73, 0.84 |
|  | 2015 | 107,303 | 904 | 0.84 | 0.79, 0.90 |
|  | 2016 | 109,612 | 1,000 | 0.91 | 0.86, 0.97 |
|  | 2017 | 111,873 | 1,098 | 0.98 | 0.93, 1.04 |
|  | 2018 | 113,968 | 1,262 | 1.11 | 1.05, 1.17 |
|  | 2019 | 116,473 | 1,435 | 1.23 | 1.17, 1.30 |
|  | 2020 | 118,549 | 1,658 | 1.40 | 1.33, 1.47 |
|  | 2021 | 119,407 | 1,908 | 1.60 | 1.53, 1.67 |
|  | 2022 | 120,499 | 2,202 | 1.83 | 1.75, 1.91 |
| **13-17 years** | 2011 | 80,088 | 541 | 0.68 | 0.62, 0.73 |
|  | 2012 | 79,614 | 569 | 0.71 | 0.66, 0.78 |
|  | 2013 | 78,852 | 597 | 0.76 | 0.70, 0.82 |
|  | 2014 | 78,325 | 611 | 0.78 | 0.72, 0.84 |
|  | 2015 | 77,877 | 649 | 0.88 | 0.77, 0.90 |
|  | 2016 | 77,790 | 707 | 0.91 | 0.84, 0.98 |
|  | 2017 | 78,160 | 767 | 0.98 | 0.91, 1.05 |
|  | 2018 | 78,069 | 785 | 1.01 | 0.94, 1.08 |
|  | 2019 | 78,353 | 817 | 1.04 | 0.97, 1.12 |
|  | 2020 | 49,129 | 826 | 1.04 | 0.98, 1.12 |
|  | 2021 | 80,788 | 900 | 1.11 | 1.04, 1.19 |
|  | 2022 | 82,613 | 971 | 1.18 | 1.10, 1.25 |

| **Appendix 1. Cont’d.**  **Crude Annual Prevalence and Incidence Rates of Autism in Manitoba Children and Adolescents, by Age Group (2011-2022)**  Numeric estimates corresponding to Figure 2 | | | | | |
| --- | --- | --- | --- | --- | --- |
| **Incidence** | | | | | |
| **Age Group** | **Year** | **Total person-years** | **Newly diagnosed autism cases** | **Autism diagnosis rate/1,000 people** | **95% CI** |
| **0-5 years** | 2011 | 57,437 | 99 | 1.72 | 1.41, 2.10 |
|  | 2012 | 59,859 | 113 | 1.89 | 1.57, 2.27 |
|  | 2013 | 61,064 | 139 | 2.28 | 1.93, 2.69 |
|  | 2014 | 61,458 | 156 | 2.54 | 2.17, 2.97 |
|  | 2015 | 61,947 | 181 | 2.92 | 2.52, 3.38 |
|  | 2016 | 62,635 | 196 | 3.13 | 2.72, 3.60 |
|  | 2017 | 63,235 | 274 | 4.33 | 3.85, 4.88 |
|  | 2018 | 64,166 | 225 | 3.51 | 3.08, 4.00 |
|  | 2019 | 64,678 | 278 | 4.14 | 3.68, 4.67 |
|  | 2020 | 65,058 | 281 | 4.32 | 3.84, 4.86 |
|  | 2021 | 64,954 | 526 | 8.10 | 7.43, 8.82 |
|  | 2022 | 64,435 | 595 | 9.23 | 8.52, 10.01 |
| **6-12 years** | 2011 | 98,897 | 59 | 0.60 | 0.46, 0.77 |
|  | 2012 | 100,210 | 46 | 0.46 | 0.34, 0.61 |
|  | 2013 | 101,575 | 55 | 0.54 | 0.42, 0.71 |
|  | 2014 | 103,900 | 60 | 0.58 | 0.45, 0.74 |
|  | 2015 | 105,667 | 75 | 0.71 | 0.57, 0.89 |
|  | 2016 | 108,209 | 69 | 0.64 | 0.50, 0.81 |
|  | 2017 | 110,009 | 72 | 0.65 | 0.52, 0.82 |
|  | 2018 | 111,857 | 48 | 0.43 | 0.32, 0.57 |
|  | 2019 | 114,224 | 54 | 0.47 | 0.36, 0.62 |
|  | 2020 | 116,331 | 75 | 0.64 | 0.51, 0.81 |
|  | 2021 | 116,711 | 96 | 0.82 | 0.67, 1.00 |
|  | 2022 | 117,513 | 111 | 0.94 | 0.78, 1.14 |
| **13-17 years** | 2011 | 79,242 | 28 | 0.35 | 0.24, 0.51 |
|  | 2012 | 78,745 | 32 | 0.41 | 0.29, 0.57 |
|  | 2013 | 77,781 | 33 | 0.42 | 0.30, 0.60 |
|  | 2014 | 77,236 | 35 | 0.45 | 0.33, 0.63 |
|  | 2015 | 76,736 | 32 | 0.42 | 0.30, 0.59 |
|  | 2016 | 76,841 | 29 | 0.38 | 0.26, 0.54 |
|  | 2017 | 76,905 | 46 | 0.60 | 0.45, 0.80 |
|  | 2018 | 76,766 | 41 | 0.53 | 0.39, 0.73 |
|  | 2019 | 77,023 | 35 | 0.45 | 0.33, 0.63 |
|  | 2020 | 78,013 | 42 | 0.54 | 0.40, 0.73 |
|  | 2021 | 79,399 | 59 | 0.74 | 0.58, 0.96 |
|  | 2022 | 81,175 | 62 | 0.76 | 0.60, 0.98 |

| **Appendix 2. Crude Annual Prevalence and Incidence Rates of Autism in Manitoba Children and Adolescents, by Sex (2011-2022)**  Numeric estimates corresponding to Figure 3 | | | | | |
| --- | --- | --- | --- | --- | --- |
| **Prevalence** | | | | | |
| **Sex** | **Year** | **Total**  **population** | **Autism cases** | **Autism prevalence (%)** | **95% CI** |
| **Male** | 2011 | 122,135 | 1,230 | 1.01 | 0.95, 1.07 |
|  | 2012 | 123,835 | 1,312 | 1.06 | 1.00, 1.12 |
|  | 2013 | 124,970 | 1,405 | 1.12 | 1.07, 1.18 |
|  | 2014 | 126,324 | 1,555 | 1.23 | 1.17, 1.29 |
|  | 2015 | 127,413 | 1,739 | 1.36 | 1.30, 1.43 |
|  | 2016 | 128,783 | 1,948 | 1.51 | 1.45, 1.58 |
|  | 2017 | 130,721 | 2,203 | 1.69 | 1.62, 1.76 |
|  | 2018 | 132,262 | 2,391 | 1.81 | 1.74, 1.88 |
|  | 2019 | 133,854 | 1,625 | 1.96 | 1.89, 2.04 |
|  | 2020 | 135,689 | 2,843 | 2.10 | 2.02, 2.17 |
|  | 2021 | 136,938 | 3,294 | 2.41 | 2.32, 2.49 |
|  | 2022 | 138,160 | 3,904 | 2.75 | 2.67, 2.84 |
| **Female** | 2011 | 115,863 | 325 | 0.28 | 0.25, 0.31 |
|  | 2012 | 117,676 | 349 | 0.30 | 0.27, 0.33 |
|  | 2013 | 118,816 | 375 | 0.32 | 0.29, 0.35 |
|  | 2014 | 119,871 | 391 | 0.33 | 0.30, 0.36 |
|  | 2015 | 120,739 | 417 | 0.35 | 0.31. 0.38 |
|  | 2016 | 122,166 | 473 | 0.39 | 0.35, 0.42 |
|  | 2017 | 123,795 | 537 | 0.43 | 0.40, 0.47 |
|  | 2018 | 125,326 | 598 | 0.48 | 0.44, 0.52 |
|  | 2019 | 127,080 | 658 | 0.52 | 0.48, 0.56 |
|  | 2020 | 128,387 | 750 | 0.58 | 0.54, 0.63 |
|  | 2021 | 129,848 | 896 | 0.69 | 0.65, 0.74 |
|  | 2022 | 131,356 | 1,102 | 0.84 | 0.79, 0.89 |

| **Appendix 2. Cont’d.**  **Crude Annual Prevalence and Incidence Rates of Autism in Manitoba Children and Adolescents, by Sex (2011-2022)**  Numeric estimates corresponding to Figure 3 | | | | | |
| --- | --- | --- | --- | --- | --- |
| **Incidence** | | | | | |
| **Sex** | **Year** | **Total person-years** | **Newly diagnosed autism cases** | **Autism diagnosis rate/1,000 people** | **95% CI** |
| **Male** | 2011 | 120,478 | 143 | 1.19 | 1.01, 1.40 |
|  | 2012 | 122,001 | 151 | 1.24 | 1.06, 1.45 |
|  | 2013 | 122,787 | 185 | 1.51 | 1.30, 1.74 |
|  | 2014 | 123,970 | 209 | 1.69 | 1.47, 1.90 |
|  | 2015 | 124,887 | 240 | 1.92 | 1.69, 2.18 |
|  | 2016 | 126,426 | 228 | 1.80 | 1.58, 2.05 |
|  | 2017 | 127,725 | 309 | 2.42 | 2.16, 2.70 |
|  | 2018 | 128,981 | 243 | 1.88 | 1.66, 2.14 |
|  | 2019 | 130,404 | 285 | 2.19 | 1.95, 2.45 |
|  | 2020 | 132,346 | 293 | 2.21 | 1.97, 2.48 |
|  | 2021 | 132,933 | 509 | 3.83 | 3.51, 4.18 |
|  | 2022 | 133,676 | 551 | 4.12 | 3.79, 4.48 |
| **Female** | 2011 | 115,098 | 43 | 0.37 | 0.28, 0.50 |
|  | 2012 | 116,814 | 40 | 0.34 | 0.25, 0.47 |
|  | 2013 | 117,632 | 42 | 0.36 | 0.26, 0.48 |
|  | 2014 | 118,623 | 42 | 0.35 | 0.26, 0.48 |
|  | 2015 | 119,462 | 48 | 0.40 | 0.30, 0.53 |
|  | 2016 | 121,258 | 66 | 0.54 | 0.43, 0.69 |
|  | 2017 | 122,424 | 83 | 0.68 | 0.55, 0.84 |
|  | 2018 | 123,808 | 71 | 0.57 | 0.45, 0.72 |
|  | 2019 | 125,521 | 72 | 0.57 | 0.46, 0.72 |
|  | 2020 | 127,056 | 105 | 0.83 | 0.68, 1.00 |
|  | 2021 | 128,130 | 172 | 1.34 | 1.16, 1.56 |
|  | 2022 | 129,447 | 217 | 1.68 | 1.47, 1.91 |

| **Appendix 3. Crude Annual Prevalence and Incidence Rates of Autism in Manitoba Children and Adolescents, by Urban and Rural Income Quintiles (2011-2022)** | | | | | |
| --- | --- | --- | --- | --- | --- |
| **Prevalence** | | | | | |
| **Group** | **Year** | **Total population** | **Autism cases** | **Autism prevalence (%)** | **95% CI** |
| **Urban Q1 (lowest)** | 2011 | 32,249 | 204 | 0.63 | 0.55, 0.73 |
|  | 2012 | 33,261 | 230 | 0.69 | 0.61, 0.79 |
|  | 2013 | 33,863 | 261 | 0.77 | 0.68, 0.87 |
|  | 2014 | 33,407 | 297 | 0.89 | 0.79, 1.00 |
|  | 2015 | 33,739 | 326 | 0.97 | 0.87, 1.08 |
|  | 2016 | 34,208 | 393 | 1.15 | 1.04, 1.27 |
|  | 2017 | 35,043 | 486 | 1.39 | 1.27, 1.52 |
|  | 2018 | 36,291 | 523 | 1.44 | 1.32, 1.57 |
|  | 2019 | 34,504 | 551 | 1.60 | 1.47. 1.74 |
|  | 2020 | 35,200 | 614 | 1.74 | 1.61, 1.89 |
|  | 2021 | 34,534 | 720 | 2.08 | 1.94, 2.24 |
|  | 2022 | 33,924 | 844 | 2.49 | 2.33, 2.66 |
| **Urban Q2** | 2011 | 28,954 | 190 | 0.66 | 0.57, 0.76 |
|  | 2012 | 29,998 | 215 | 0.72 | 0.63, 0.82 |
|  | 2013 | 29,967 | 232 | 0.77 | 0.68, 0.88 |
|  | 2014 | 32,285 | 285 | 0.88 | 0.79, 0.99 |
|  | 2015 | 32,962 | 349 | 1.06 | 0.95, 1.18 |
|  | 2016 | 33,373 | 389 | 1.17 | 1.06, 1.29 |
|  | 2017 | 34,524 | 451 | 1.31 | 1.19, 1.43 |
|  | 2018 | 34,314 | 481 | 1.40 | 1.28, 1.53 |
|  | 2019 | 34,736 | 541 | 1.56 | 1.43, 1.69 |
|  | 2020 | 33,497 | 605 | 1.81 | 1.67, 1.96 |
|  | 2021 | 34,078 | 738 | 2.17 | 2.01, 2.33 |
|  | 2022 | 32,779 | 835 | 2.55 | 2.38, 2.73 |
| **Urban Q3** | 2011 | 30,929 | 248 | 0.80 | 0.71, 0.91 |
|  | 2012 | 31,590 | 254 | 0.80 | 0.55, 0.73 |
|  | 2013 | 31,957 | 278 | 0.87 | 0.61, 0.79 |
|  | 2014 | 29,980 | 274 | 0.91 | 0.68, 0.87 |
|  | 2015 | 30,259 | 318 | 1.05 | 0.79, 1.00 |
|  | 2016 | 30,599 | 347 | 1.13 | 0.87, 1.08 |
|  | 2017 | 30,925 | 380 | 1.23 | 1.04, 1.27 |
|  | 2018 | 31,509 | 419 | 1.33 | 1.27, 1.52 |
|  | 2019 | 33,525 | 451 | 1.35 | 1.32, 1.57 |
|  | 2020 | 33,175 | 518 | 1.56 | 1.47, 1.74 |
|  | 2021 | 33,183 | 618 | 1.86 | 1.61, 1.89 |
|  | 2022 | 33,119 | 709 | 2.14 | 1.94, 2.24 |
| **Urban Q4** | 2011 | 30,158 | 219 | 0.73 | 2.33, 2.66 |
|  | 2012 | 30,485 | 229 | 0.75 | 0.57, 0.76 |
|  | 2013 | 30,774 | 246 | 0.80 | 0.63, 0.82 |
|  | 2014 | 31,192 | 267 | 0.86 | 0.68, 0.88 |
|  | 2015 | 31,310 | 286 | 0.91 | 0.79, 0.99 |
|  | 2016 | 31,567 | 318 | 1.01 | 0.95, 1.18 |
|  | 2017 | 32,771 | 347 | 1.06 | 1.06, 1.29 |
|  | 2018 | 33,044 | 367 | 1.11 | 1.19, 1.4 |
|  | 2019 | 32,677 | 403 | 1.23 | 1.28, 1.53 |
|  | 2020 | 33,658 | 449 | 1.33 | 1.431, 1.69 |
|  | 2021 | 33,018 | 512 | 1.55 | 1.67, 1.96 |
|  | 2022 | 33,604 | 592 | 1.76 | 2.01, 2.33 |
| **Urban Q5 (highest)** | 2011 | 33,008 | 213 | 0.65 | 2.38, 2.73 |
|  | 2012 | 33,306 | 228 | 0.68 | 0.71, 0.91 |
|  | 2013 | 33,210 | 239 | 0.72 | 0.55, 0.73 |
|  | 2014 | 33,811 | 258 | 0.76 | 0.61, 0.79 |
|  | 2015 | 34,168 | 280 | 0.82 | 0.73, 0.92 |
|  | 2016 | 34,508 | 301 | 0.87 | 0.78, 0.98 |
|  | 2017 | 34,432 | 332 | 0.96 | 0.87, 1.07 |
|  | 2018 | 34,400 | 352 | 1.02 | 0.92, 1.14 |
|  | 2019 | 33,947 | 360 | 1.06 | 0.96, 1.18 |
|  | 2020 | 34,045 | 387 | 1.14 | 1.03, 1.26 |
|  | 2021 | 33,365 | 425 | 1.27 | 1.16, 1.40 |
|  | 2022 | 34,095 | 510 | 1.50 | 1.37, 1.63 |
| **Rural Q1 (lowest)** | 2011 | 32,249 | 105 | 0.33 | 0.27, 0.39 |
|  | 2012 | 32,123 | 99 | 0.31 | 0.25, 0.38 |
|  | 2013 | 31,867 | 105 | 0.33 | 0.27, 0.40 |
|  | 2014 | 32,357 | 125 | 0.39 | 0.32, 0.46 |
|  | 2015 | 32,807 | 144 | 0.44 | 0.37, 0.52 |
|  | 2016 | 32,319 | 163 | 0.50 | 0.43, 0.59 |
|  | 2017 | 31,755 | 183 | 0.58 | 0.50, 0.67 |
|  | 2018 | 32,499 | 214 | 0.66 | 0.58, 0.75 |
|  | 2019 | 26,381 | 194 | 0.74 | 0.64, 0.85 |
|  | 2020 | 28,281 | 218 | 0.77 | 0.68, 0.88 |
|  | 2021 | 28,384 | 269 | 0.95 | 0.84, 1.07 |
|  | 2022 | 28,133 | 325 | 1.16 | 1.04, 1.29 |
| **Rural Q2** | 2011 | 23,355 | 86 | 0.37 | 0.30, 0.45 |
|  | 2012 | 23,533 | 94 | 0.40 | 0.33, 0.49 |
|  | 2013 | 23,680 | 102 | 0.43 | 0.35, 0.52 |
|  | 2014 | 26,327 | 143 | 0.54 | 0.46, 0.64 |
|  | 2015 | 26,127 | 161 | 0.62 | 0.53, 0.72 |
|  | 2016 | 26,131 | 177 | 0.68 | 0.58, 0.78 |
|  | 2017 | 27,228 | 210 | 0.77 | 0.67, 0.88 |
|  | 2018 | 26,652 | 222 | 0.83 | 0.73, 0.95 |
|  | 2019 | 31,775 | 257 | 0.81 | 0.72, 0.91 |
|  | 2020 | 30,067 | 269 | 0.89 | 0.79, 1.01 |
|  | 2021 | 30,768 | 327 | 1.06 | 0.95, 1.18 |
|  | 2022 | 30,932 | 363 | 1.17 | 1.06. 1.30 |
| **Rural Q3** | 2011 | 22,154 | 111 | 0.50 | 0.42, 0.60 |
|  | 2012 | 22,338 | 118 | 0.53 | 0.44, 0.63 |
|  | 2013 | 22,202 | 124 | 0.56 | 0.47, 0.67 |
|  | 2014 | 21,989 | 115 | 0.52 | 0.44, 0.63 |
|  | 2015 | 22,545 | 124 | 0.55 | 0.46, 0.66 |
|  | 2016 | 22,897 | 142 | 0.62 | 0.53, 0.73 |
|  | 2017 | 23,679 | 158 | 0.67 | 0.57, 0.78 |
|  | 2018 | 23,941 | 182 | 0.76 | 0.66, 0.88 |
|  | 2019 | 25,620 | 214 | 0.84 | 0.73, 0.96 |
|  | 2020 | 25,882 | 217 | 0.84 | 0.73, 0.96 |
|  | 2021 | 25,522 | 253 | 0.99 | 0.88, 1.12 |
|  | 2022 | 25,692 | 297 | 1.16 | 1.03, 1.30 |
| **Rural Q4** | 2011 | 26,642 | 107 | 0.40 | 0.33, 0.49 |
|  | 2012 | 27,125 | 118 | 0.44 | 0.36, 0.52 |
|  | 2013 | 27,648 | 143 | 0.52 | 0.44, 0.61 |
|  | 2014 | 23,909 | 124 | 0.52 | 0.43, 0.62 |
|  | 2015 | 24,190 | 114 | 0.47 | 0.39, 0.57 |
|  | 2016 | 24,902 | 140 | 0.56 | 0.48, 0.66 |
|  | 2017 | 25,496 | 159 | 0.62 | 0.53, 0.73 |
|  | 2018 | 25,983 | 163 | 0.63 | 0.54, 0.73 |
|  | 2019 | 25,640 | 226 | 0.88 | 0.77, 1.00 |
|  | 2020 | 25,821 | 237 | 0.92 | 0.81, 1.04 |
|  | 2021 | 25,856 | 265 | 1.02 | 0.91, 1.16 |
|  | 2022 | 26,867 | 293 | 1.09 | 0.97, 1.22 |
| **Rural Q5 (highest)** | 2011 | 23,362 | 124 | 0.53 | 0.45, 0.63 |
|  | 2012 | 23,802 | 132 | 0.55 | 0.47, 0.66 |
|  | 2013 | 23,993 | 128 | 0.53 | 0.45, 0.63 |
|  | 2014 | 25,792 | 150 | 0.58 | 0.50, 0.68 |
|  | 2015 | 25,149 | 166 | 0.66 | 0.57, 0.77 |
|  | 2016 | 25,525 | 173 | 0.68 | 0.58, 0.79 |
|  | 2017 | 24,302 | 184 | 0.76 | 0.66, 0.87 |
|  | 2018 | 24,438 | 199 | 0.81 | 0.71, 0.94 |
|  | 2019 | 25,155 | 203 | 0.81 | 0.70, 0.93 |
|  | 2020 | 26,258 | 217 | 0.83 | 0.72, 0.94 |
|  | 2021 | 26,986 | 273 | 1.01 | 0.90, 1.14 |
|  | 2022 | 27,816 | 316 | 1.14 | 1.02, 1.27 |


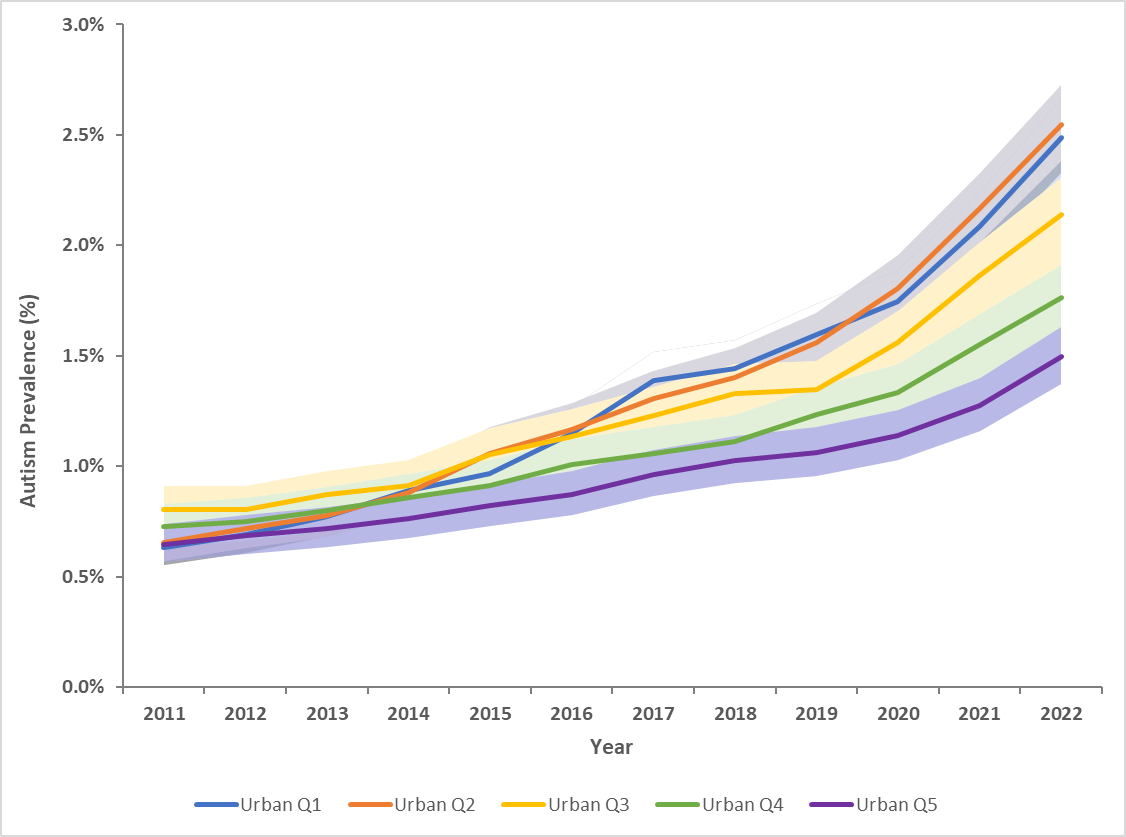


**Crude Annual Prevalence Rates of Autism in Manitoba Children and Adolescents, by Urban Income Quintiles (2011-2022)**


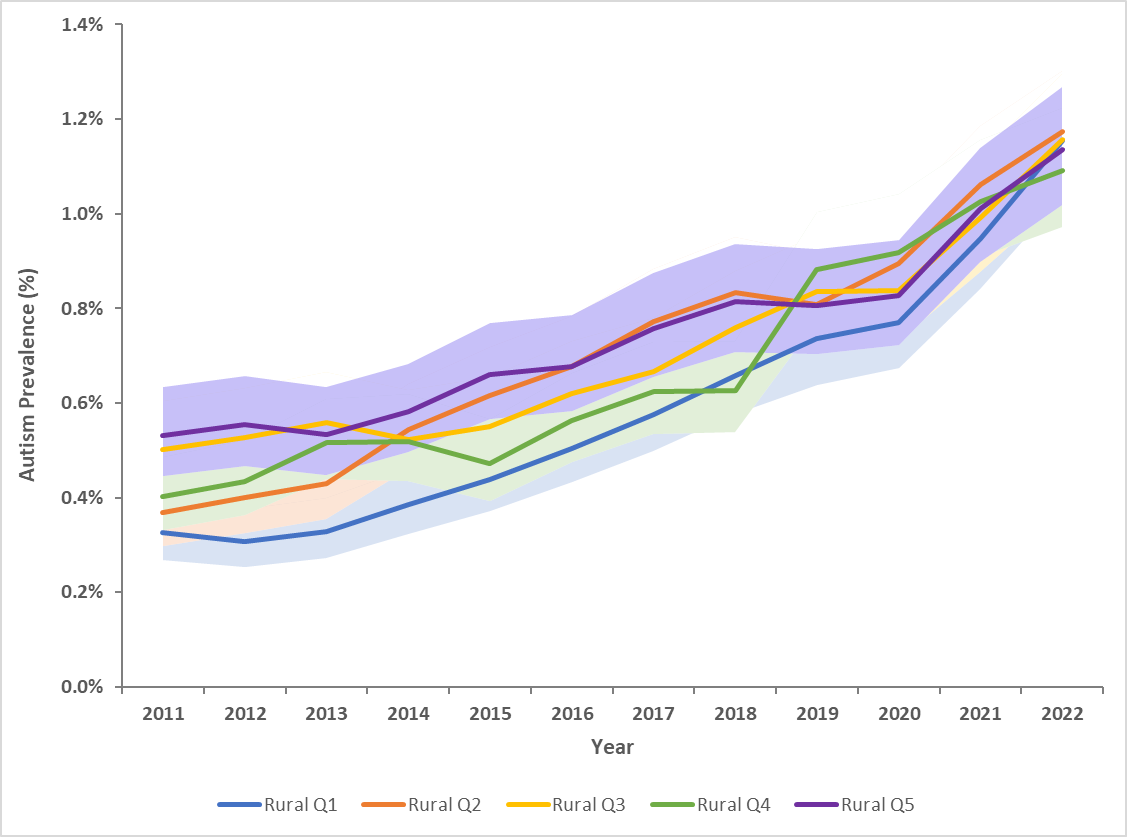


**Crude Annual Prevalence Rates of Autism in Manitoba Children and Adolescents, by Rural Income Quintiles (2011-2022)**

| **Appendix 3. Cont’d.**  **Crude Annual Prevalence and Incidence Rates of Autism in Manitoba Children and Adolescents, by Urban and Rural Income Quintiles (2011-2022)** | | | | | |
| --- | --- | --- | --- | --- | --- |
| **Incidence** | | | | | |
| **Group** | **Year** | **Total person-years** | **Newly diagnosed autism cases** | **Autism incidence rate/1,000 people** | **95% CI** |
| **Urban Q1 (lowest)** | 2011 | 31,897 | 36 | 1.13 | 0.81, 1.56 |
|  | 2012 | 32,752 | 50 | 1.53 | 1.16, 2.01 |
|  | 2013 | 33,247 | 52 | 1.56 | 1.19, 2.05 |
|  | 2014 | 32,720 | 59 | 1.80 | 1.40, 2.33 |
|  | 2015 | 33,053 | 64 | 1.94 | 1.52, 2.47 |
|  | 2016 | 33,574 | 77 | 2.29 | 1.83, 2.87 |
|  | 2017 | 34,207 | 118 | 3.45 | 2.88, 4.13 |
|  | 2018 | 35,272 | 82 | 2.32 | 1.87, 2.89 |
|  | 2019 | 33,572 | 88 | 2.62 | 2.13, 3.23 |
|  | 2020 | 34,313 | 100 | 2.91 | 2.40, 3.55 |
|  | 2021 | 33,497 | 166 | 4.96 | 4.26, 5.77 |
|  | 2022 | 32,776 | 168 | 5.13 | 4.41, 5.96 |
| **Urban Q2** | 2011 | 28,649 | 24 | 0.84 | 0.56, 1.25 |
|  | 2012 | 29,625 | 27 | 0.91 | 0.63, 1.33 |
|  | 2013 | 29,473 | 48 | 1.63 | 1.23, 2.16 |
|  | 2014 | 31,750 | 52 | 1.64 | 1.25, 2.15 |
|  | 2015 | 32,371 | 79 | 2.44 | 1.96, 3.04 |
|  | 2016 | 32,851 | 67 | 2.04 | 1.61, 2.59 |
|  | 2017 | 33,820 | 66 | 1.95 | 1.53, 2.48 |
|  | 2018 | 33,491 | 65 | 1.94 | 1.52, 2.48 |
|  | 2019 | 33,912 | 83 | 2.45 | 1.97, 3.04 |
|  | 2020 | 32,718 | 92 | 2.81 | 2.29, 3.45 |
|  | 2021 | 33,124 | 152 | 4.59 | 3.91, 5.38 |
|  | 2022 | 31,758 | 162 | 5.10 | 4.37, 5.95 |
| **Urban Q3** | 2011 | 30,568 | 38 | 1.24 | 0.90, 1.71 |
|  | 2012 | 31,142 | 19 | 0.61 | 0.39, 0.96 |
|  | 2013 | 31,431 | 44 | 1.40 | 1.04, 1.88 |
|  | 2014 | 29,444 | 40 | 1.36 | 1.00, 1.85 |
|  | 2015 | 29,711 | 48 | 1.62 | 1.22, 2.14 |
|  | 2016 | 30,130 | 43 | 1.43 | 1.06, 1.92 |
|  | 2017 | 30,307 | 60 | 1.98 | 1.54, 2.55 |
|  | 2018 | 30,782 | 49 | 1.59 | 1.20, 2.11 |
|  | 2019 | 32,822 | 54 | 1.65 | 1.26, 2.15 |
|  | 2020 | 32,541 | 81 | 2.49 | 2.00, 3.09 |
|  | 2021 | 32,381 | 112 | 3.46 | 2.87, 4.16 |
|  | 2022 | 32,225 | 123 | 3.82 | 3.20, 4.55 |
| **Urban Q4** | 2011 | 29,839 | 25 | 0.84 | 0.57, 1.24 |
|  | 2012 | 30,153 | 36 | 1.19 | 0.86, 1.66 |
|  | 2013 | 30,287 | 30 | 0.99 | 0.69, 1.42 |
|  | 2014 | 30,688 | 34 | 1.11 | 0.79, 1.55 |
|  | 2015 | 30,753 | 38 | 1.24 | 0.90, 1.70 |
|  | 2016 | 31,098 | 40 | 1.29 | 0.94, 1.75 |
|  | 2017 | 32,158 | 54 | 1.68 | 1.29, 2.1 |
|  | 2018 | 32,367 | 41 | 1.27 | 0.93, 1.72 |
|  | 2019 | 31,989 | 32 | 1.00 | 0.71, 1.41 |
|  | 2020 | 33,004 | 55 | 1.67 | 1.28, 2.17 |
|  | 2021 | 32,247 | 95 | 2.95 | 2.41, 3.60 |
|  | 2022 | 32,762 | 103 | 3.14 | 2.59, 3.81 |
| **Urban Q5 (highest)** | 2011 | 32,679 | 19 | 0.58 | 0.37, 0.91 |
|  | 2012 | 32,919 | 27 | 0.82 | 0.56, 1.20 |
|  | 2013 | 32,706 | 25 | 0.76 | 0.52, 1.13 |
|  | 2014 | 33,262 | 36 | 1.08 | 0.78, 1.50 |
|  | 2015 | 33,606 | 32 | 0.95 | 0.67, 1.35 |
|  | 2016 | 34,037 | 28 | 0.82 | 0.57, 1.19 |
|  | 2017 | 33,783 | 51 | 1.51 | 1.15, 1.99 |
|  | 2018 | 33,624 | 43 | 1.28 | 0.95, 1.72 |
|  | 2019 | 33,329 | 39 | 1.17 | 0.85, 1.60 |
|  | 2020 | 33,500 | 46 | 1.37 | 1.03, 1.83 |
|  | 2021 | 32,683 | 66 | 2.02 | 1.59, 2.57 |
|  | 2022 | 33,363 | 87 | 2.61 | 2.11, 3.22 |
| **Rural Q1 (lowest)** | 2011 | 32,011 | 21 | 0.66 | 0.43, 1.01 |
|  | 2012 | 31,889 | 12 | 0.38 | 0.21, 0.66 |
|  | 2013 | 31,559 | 16 | 0.51 | 0.31, 0.83 |
|  | 2014 | 32,013 | 31 | 0.97 | 0.68, 1.38 |
|  | 2015 | 32,440 | 24 | 0.74 | 0.50, 1.10 |
|  | 2016 | 32,045 | 32 | 1.00 | 0.71, 1.41 |
|  | 2017 | 31,401 | 47 | 1.50 | 1.12, 1.99 |
|  | 2018 | 32,069 | 38 | 1.18 | 0.86, 1.63 |
|  | 2019 | 26,020 | 37 | 1.42 | 1.03, 1.96 |
|  | 2020 | 27,940 | 32 | 1.15 | 0.81, 1.62 |
|  | 2021 | 27,934 | 62 | 2.22 | 1.73, 2.85 |
|  | 2022 | 27,655 | 61 | 2.21 | 1.72, 2.84 |
| **Rural Q2** | 2011 | 23,158 | 9 | 0.39 | 0.20, 0.75 |
|  | 2012 | 23,289 | 10 | 0.43 | 0.23, 0.80 |
|  | 2013 | 23,391 | 15 | 0.64 | 0.39, 1.06 |
|  | 2014 | 26,003 | 21 | 0.81 | 0.53, 1.24 |
|  | 2015 | 25,799 | 20 | 0.78 | 0.50, 1.20 |
|  | 2016 | 25,870 | 23 | 0.89 | 0.59, 1.34 |
|  | 2017 | 26,844 | 29 | 1.08 | 0.75, 1.55 |
|  | 2018 | 26,248 | 25 | 0.95 | 0.64, 1.41 |
|  | 2019 | 31,309 | 34 | 1.09 | 0.78, 1.52 |
|  | 2020 | 29,686 | 33 | 1.11 | 0.79, 1.56 |
|  | 2021 | 30,289 | 54 | 1.78 | 1.37, 2.33 |
|  | 2022 | 30,422 | 67 | 2.20 | 1.73, 2.80 |
| **Rural Q3** | 2011 | 21,963 | 15 | 0.68 | 0.41, 1.13 |
|  | 2012 | 22,108 | 14 | 0.63 | 0.38, 1.07 |
|  | 2013 | 21,923 | 19 | 0.87 | 0.55, 1.36 |
|  | 2014 | 21,718 | 13 | 0.60 | 0.35, 1.03 |
|  | 2015 | 22,244 | 18 | 0.81 | 0.51, 1.28 |
|  | 2016 | 22,650 | 26 | 1.15 | 0.78, 1.69 |
|  | 2017 | 23,329 | 24 | 1.03 | 0.69, 1.53 |
|  | 2018 | 23,566 | 29 | 1.23 | 0.86, 1.77 |
|  | 2019 | 25,243 | 26 | 1.03 | 0.70, 1.51 |
|  | 2020 | 25,572 | 26 | 1.02 | 0.69, 1.49 |
|  | 2021 | 25,115 | 64 | 2.55 | 1.99, 3.26 |
|  | 2022 | 25,259 | 53 | 2.10 | 1.60, 2.75 |
| **Rural Q4** | 2011 | 26,431 | 15 | 0.57 | 0.34, 0.94 |
|  | 2012 | 26,875 | 16 | 0.60 | 0.36, 0.97 |
|  | 2013 | 27,333 | 23 | 0.84 | 0.56, 1.27 |
|  | 2014 | 23,614 | 12 | 0.51 | 0.29, 0.89 |
|  | 2015 | 23,885 | 11 | 0.46 | 0.26, 0.83 |
|  | 2016 | 24,668 | 24 | 0.97 | 0.65, 1.45 |
|  | 2017 | 25,177 | 23 | 0.91 | 0.61, 1.37 |
|  | 2018 | 25,629 | 15 | 0.59 | 0.35, 0.97 |
|  | 2019 | 25,247 | 38 | 1.51 | 1.10, 2.07 |
|  | 2020 | 25,465 | 22 | 0.86 | 0.57, 1.31 |
|  | 2021 | 25,440 | 44 | 1.73 | 1.29, 2.32 |
|  | 2022 | 26,429 | 46 | 1.74 | 1.30, 2.32 |
| **Rural Q5 (highest)** | 2011 | 23,163 | 16 | 0.69 | 0.42, 1.13 |
|  | 2012 | 23,555 | 14 | 0.59 | 0.35, 1.00 |
|  | 2013 | 23,664 | 12 | 0.51 | 0.29, 0.89 |
|  | 2014 | 25,459 | 18 | 0.71 | 0.45, 1.12 |
|  | 2015 | 24,813 | 32 | 1.29 | 0.91, 1.82 |
|  | 2016 | 25,246 | 19 | 0.75 | 0.48, 1.18 |
|  | 2017 | 23,960 | 32 | 1.34 | 0.94, 1.89 |
|  | 2018 | 24,082 | 18 | 0.75 | 0.47, 1.19 |
|  | 2019 | 24,808 | 15 | 0.60 | 0.36, 1.00 |
|  | 2020 | 25,957 | 25 | 0.96 | 0.65, 1.43 |
|  | 2021 | 26,586 | 44 | 1.66 | 1.23, 2.22 |
|  | 2022 | 27,380 | 43 | 1.57 | 1.16, 2.12 |


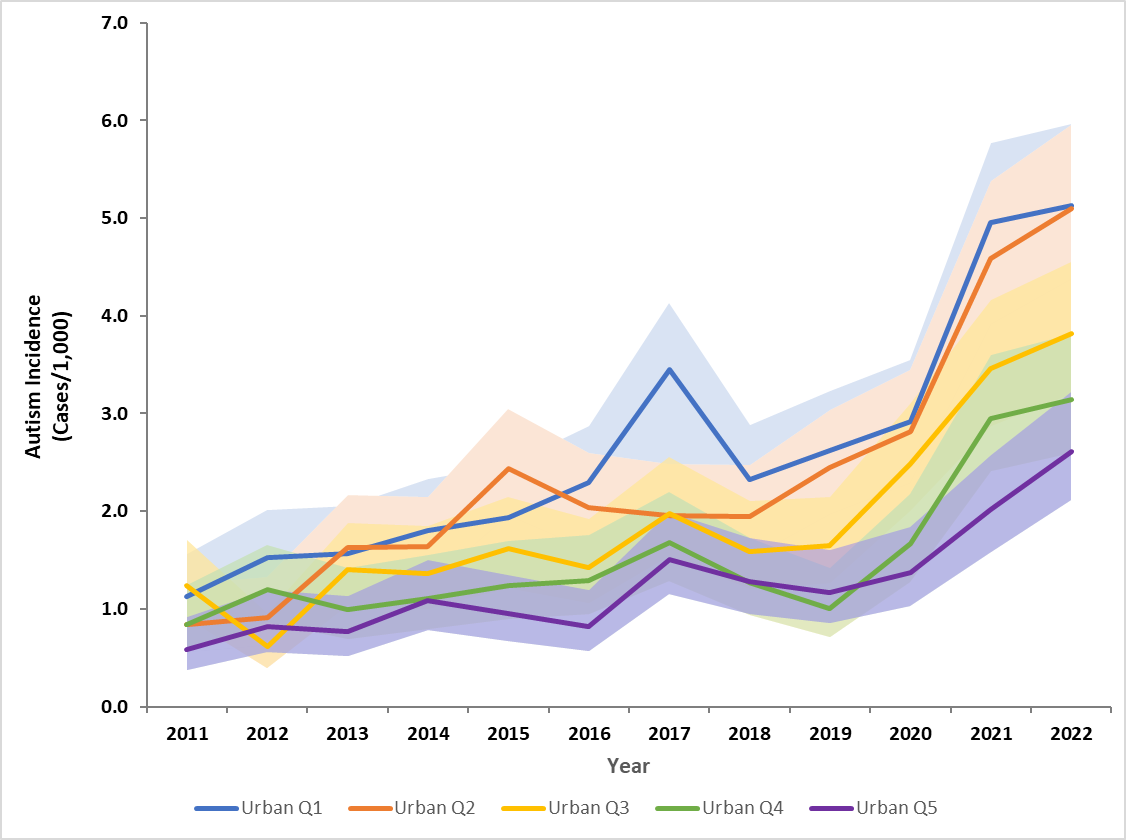


**Crude Annual Incidence Rates of Autism in Manitoba Children and Adolescents, by Urban Income Quintiles (2011-2022)**


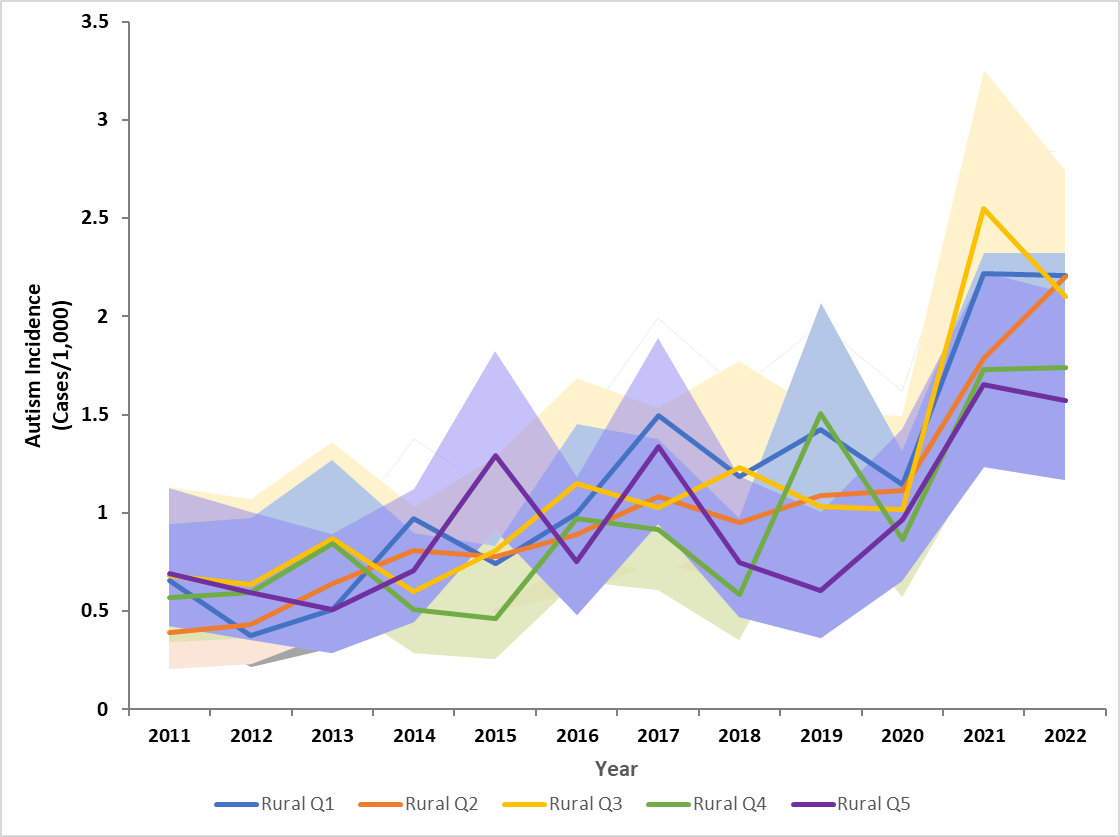


**Crude Annual Incidence Rates of Autism in Manitoba Children and Adolescents, by Rural Income Quintiles (2011-2022)**

| **Appendix 4. Annual Trends in Autism Prevalence and Incidence in Manitoba from 2011-2022**  Children and adolescents aged 0-17 years, by urban and rural income quintiles | | | |
| --- | --- | --- | --- |
| **Prevalence** | | | |
|  |  | **Estimate** | **95% CI** |
| **Overall** | Relative rate (difference each year) | 1.103 | 1.100, 1.107 |
|  |  |  |  |
| **By urban income quintile** | Relative rate (difference each year) | 1.106 | 1.102, 1.110 |
|  | Urban Q1 | 1.384 | 1.328, 1.442 |
|  | Urban Q2 | 1.403 | 1.328, 1.442 |
|  | Urban Q3 | 1.312 | 1.258, 1.369 |
|  | Urban Q4 | 1.141 | 1.093, 1.192 |
|  | Urban Q5 (ref group) | 1.000 | 1.000, 1.000 |
|  |  |  |  |
| **By rural income quintile** | Relative rate (difference each year) | 1.098 | 1.092, 1.104 |
|  | Rural Q1 | 0.801 | 0.755, 0.849 |
|  | Rural Q2 | 0.969 | 0.915, 1.027 |
|  | Rural Q3 | 0.960 | 0.904, 1.019 |
|  | Rural Q4 | 0.912 | 0.860, 0.968 |
|  | Rural Q5 (ref group) | 1.000 | 1.000, 1.000 |
| **Incidence** | | | |
|  |  | **Estimate** | **95% CI** |
| **Overall** | Relative rate (difference each year) | 1.123 | 1.097, 1.149 |
|  |  |  |  |
| **By urban income quintile** | Relative rate (difference each year) | 1.125 | 1.108, 1.143 |
|  | Urban Q1 | 2.096 | 1.774, 2.475 |
|  | Urban Q2 | 1.854 | 1.567, 2.195 |
|  | Urban Q3 | 1.503 | 1.265, 1.786 |
|  | Urban Q4 | 1.230 | 1.031, 1.466 |
|  | Urban Q5 (ref group) | 1.000 | 1.000, 1.000 |
|  |  |  |  |
| **By rural income quintile** | Relative rate (difference each year) | 1.120 | 1.099, 1.141 |
|  | Rural Q1 | 1.187 | 0.972, 1.450 |
|  | Rural Q2 | 1.041 | 0.846, 1.280 |
|  | Rural Q3 | 1.177 | 0.957, 1.448 |
|  | Rural Q4 | 0.979 | 0.793, 1.208 |
|  | Rural Q5 (ref group) | 1.000 | 1.000, 1.000 |
